# The Plant of Many Scents: Unraveling the Odorant Composition of Selected CBD Hemp Cultivars

**DOI:** 10.1021/acs.jafc.5c07208

**Published:** 2025-09-10

**Authors:** Thi Khanh Linh Tran, Tatiana Avellaneda, Amandine André, Elodie Gillich, Martin Steinhaus, Dániel Árpád Carrera, Leron Katsir, Irene Chetschik

**Affiliations:** 1 Life Sciences and Facility Management, 111833 Zurich University of Applied Sciences (ZHAW), Wädenswil 8820, Switzerland; 2 Puregene AG, Zeiningen 4314, Switzerland; 3 Leibniz Institute for Food Systems Biology at the Technical University of Munich (Leibniz-LSB@TUM), Freising 85354, Germany

**Keywords:** dried hemp flowers, odor-active compounds, gas chromatography-olfactometry (GC-O), aroma extract dilution analysis (AEDA), flavor dilution factor (FD factor), *Cannabis sativa* L.

## Abstract

This study presents the first comprehensive sensory-guided investigation into the odor-active compounds of dried hemp (*Cannabis sativa* L.) flowers. Using gas chromatography-olfactometry (GC-O) in combination with aroma extract dilution analysis (AEDA), 52 odor-active compounds were identified across six cannabidiol-rich cultivars. Among them, 38 odorants were reported for the first time in dried hemp flowers, whereas six compounds have not been identified in any hemp material before. Terpenes and terpenoids such as α-pinene, myrcene, and linalool exhibited consistently high flavor dilution (FD) factors of 256–1024 across all cultivars, suggesting their role as important contributors to hemp aroma beyond their known abundance. In addition, potent sulfur-containing compounds, including 3-methylbut-2-ene-1-thiol, 4-methyl-4-sulfanylpentan-2-one, 3-sulfanylhexan-1-ol, and 3-sulfanylhexyl acetate, were detected at high FD factors (FD 256–1024) in dried hemp flowers for the first time, confirming their sensory relevance. Other key compounds such as *p*-cresol (FD 256–1024), eugenol (FD 1024), 2-methoxy-4-vinylphenol (FD 256), methyl anthranilate (FD 256), Furaneol (FD 128), and sotolon (FD 512) were detected with high FD factors in specific cultivars, highlighting their distinct aroma characteristics. This research lays the first groundwork for understanding the odorant composition of dried hemp flowers, providing a basis for future validation through quantitation and aroma reconstitution studies.

## Graphical abstract

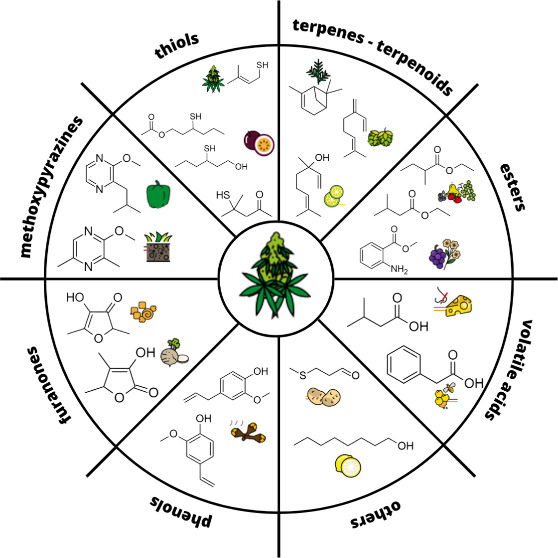

## Introduction

Hemp (*Cannabis sativa* L.) belongs to the Cannabaceae family, which includes other aromatic plants such as hops (*Humulus lupulus* L.).^1^ Hemp is rich in phytochemicals, including phytocannabinoids such as tetrahydrocannabinol (THC) and cannabidiol (CBD), as well as terpenoids, flavonoids, and sterols, all of which contribute to its biological activity and sensory properties.^2^ Traditionally, hemp was cultivated primarily for textiles and food sources due to the rich fiber content of its stems and the high oil content in its seeds.^3,4^ The inflorescence of hemp gained attention as a source of the nonpsychoactive CBD only in recent decades. The legal definition of hemp varies by country and is rooted in the context of historical prohibition. For instance, in the United States, hemp is legally defined as *Cannabis sativa* containing less than 0.3% THC by weight,^5^ whereas in Switzerland, hemp is classified with a maximum allowable THC content of 1% by weight.^6^ Due to global prohibition and regulatory restrictions throughout the 20th century,^7^ scientific exploration of hemp has been limited. In recent years, however, the expanding legalization of medicinal and recreational cannabis has renewed interest in hemp - particularly in its characteristic aroma - and has prompted more detailed studies into the volatile compounds that shape its sensory characteristics.

Aroma, rather than cannabinoids such as CBD or THC, has been shown to be the strongest predictor of consumer appeal in cannabis, highlighting its importance in perceived product quality.^8^ Consequently, aroma is increasingly prioritized as a key selection trait by breeders of both high-THC and CBD-dominant hemp cultivars. Terpenes have long been associated with the aroma of hemp, exhibiting similar volatile profiles to hops.^4^ However, recent research^9–11^ suggests that compounds from other substance classes such as sulfur-containing compounds – as well as lipid degradation products, methoxypyrazines and esters also play a crucial role in shaping the aroma of cannabis. A gas chromatography-olfactometry (GC-O) analysis on different cultivars of fresh fiber-type hemp flowers revealed 33 odor-active compounds, which includes not only terpenes but also other substance classes: lipid degradation products, methoxypyrazines, esters and sulfur-containing compounds such as 3-methylbut-2-ene-1-thiol and 4-methyl-4-sulfanylpentan-2-one.^12^ The presence of two latter compounds was subsequently confirmed in cannabis rosin extracts^9^ using comprehensive two-dimensional gas chromatography (GC × GC) analysis. This study further suggested the existence of additional prenylated volatile sulfur compounds, which were assumed as important for the characteristic sulfurous odor of cannabis rosin extract obtained from mechanically separated trichomes. In a follow-up investigation, the same group reported various esters, anthranilates, indoles, and thiols, further emphasizing the complexity of cannabis volatile fraction.^9,10^ However, the true impact of these detected volatiles on the overall cannabis aroma has not yet been investigated.

Due to the fact that only a small fraction of volatiles contribute to a product’s overall aroma, sensory-guided methods such as GC-O combined with aroma extract dilution analysis (AEDA)^13,14^ is essential in determining the relevance of volatile compounds in hemp. This approach integrates analytical techniques with human odor perception and has been extensively applied to identify key aroma compounds in various food raw materials and spices.^15–17^ AEDA analysis is performed by stepwise dilution of the volatile fraction and evaluating each dilution by GC-O, until no odorant is perceivable at the sniffing port. The flavor dilution (FD) factor refers to the highest dilution at which the compound can be smelled, revealing the first insight into the contribution of the odorant to the overall aroma impression. By means of this methodology, it was proven that only a small fraction of the volatiles contributes to the overall aroma perception of foods and food raw materials.^18^ For example for hops, the GC-O AEDA revealed that only 23 volatile compounds are odor-active in the FD factor range of 16–1024.^19^

However, application of GC-O in combination with AEDA has not been applied to dried hemp flowers and products thereof. To date, only odor-active compounds in fresh hemp flower^12^ and high-THC cannabis flowers^20^ were assessed by GC-O, leaving the odor-active constituents of dried flowers largely unexplored. In addition, mostly THC-containing materials such as rosin extracts and flowers have been previously analyzed, leaving hemp underexplored in terms of their volatile composition and the characterization of the most important contributors to the overall scent of dried cannabis flowers.

To address the aforementioned gaps, this study aimed to investigate the key odorant composition of dried hemp flowers - selectively bred for enriched CBD content and appealing aroma - using gas chromatography-olfactometry (GC-O) combined with aroma extract dilution analysis (AEDA). Terpene quantitation was additionally performed to provide a preliminary comparison with AEDA results. This represents the first application of AEDA to cannabis, which sought to identify the important molecular drivers, beyond terpenes and terpenoids, underlying the distinct aroma characteristics of different dried hemp cultivars and to gain the first insights into their contribution to the overall odor perception.

## Materials and Methods

### Hemp Samples

Freeze-dried hemp flowers of six cultivars, namely: PG071, PG072, PG073, PG074, PG075.1, and PG076 were provided by Puregene AG (Zeiningen, Switzerland). Growth conditions were standard to produce commercial hemp flower. The hemp flowers samples were harvested at optimal maturity, freeze-dried under the same condition and preserved at – 18 °C in sealed mylar bags (3.5 mil PET/aluminum laminate) prior to analyzing. Other details are provided in the Supporting Information (Table S1).

Based on qualitative sensory observations provided by professional cannabis breeder, hemp cultivars were selected for analysis based on their distinct odor profiles. PG071 presented a fruity and creamy aroma, while PG072 featured a turpentine-like odor and spicy undertones. PG073 was described as intense in earthy and herbal notes. The PG074 and PG075.1 cultivars were fruity and strawberry-like, with tropical undertones. PG076 had a citrus and turpentine-like scent.

### Reference Odorants

The reference odorants ethyl propanoate, α-pinene, methyl 3-methylbutanoate, ethyl 2-methylbutanoate, ethyl 3-methylbutanoate, 3-methyl-2-but-1-enethiol, β-pinene, myrcene, limonene, 1,8-cineole, β-phellandrene, 3-methylbutyl 2-methylbutanoate, octanal, 4-methyl-4-sulfanylpentan-2-one, 3-sulfanylhexyl acetate, 3-sulfanylhexan-1-ol, 3-methoxy-2,5-dimethylpyrazine, acetic acid, 3-(methylsulfanyl)­propanal, 3-isobutyl-2-methoxypyrazine, linalool, octan-1-ol, β-caryophyllene, 2-acetylpyrazine, butanoic acid, α-humulene, 2-methylbutanoic acid, 3-methylbutanoic acid, isoborneol, α-terpineol, (2*E*)-undec-2-enal, β-citronellol, nerol, hexanoic acid, 2-methoxyphenol, (2*E,*4*E,*6*Z*)-nona-2,4,6-trienal, (*E*)-β-ionone, 4-hydroxy-2,5-dimethylfuran-3­(2*H*)-one, 4-methylphenol, 4-allyl-2-methoxyphenol, 2-methoxy-4-vinylphenol, 3-hydroxy-4,5-dimethylfuran-2­(5*H*)-one, methyl anthranilate, indole, 3-methyl-1*H*-indole, and phenylacetic acid were purchased from Sigma-Aldrich (Buchs, Switzerland); *trans*-4,5-epoxy-(2*E)*-dec-2-enal and 2-acetyl-1-pyrroline were purchased from AromaLAB (Planegg, Germany).

### Other Chemicals and Materials

Diethyl ether (Merck KGaA) was freshly distilled before use. Anhydrous sodium sulfate was purchased from Carl Roth (Roth AG, Arlesheim, Switzerland). Methyl nonanoate was purchased from Sigma-Aldrich (Buchs, Switzerland).

### Gas Chromatography-Olfactometry (GC-O) and Aroma Extraction Dilution Analysis (AEDA)

To screen for key odorants, 10 g of dried hemp flowers were frozen with liquid nitrogen and ground finely prior to being weighed into 250 mL Erlenmeyer flasks. All ground hemp flowers were extracted with 150 mL diethyl ether by vigorous stirring with a magnetic stirrer (IKA-Werke GmbH & Co. KG, Staufen, Germany) at room temperature (20 ± 2 °C) for 3 h. During extraction, the flasks were sealed with stoppers and covered with aluminum foil. After extraction, the diethyl ether phase was filtered through filter paper (185 mm, Whatman, Germany), then directly subjected to solvent-assisted flavor evaporation (SAFE) with instrumental settings as previously described.^15^ The thawed distillates were dehydrated using anhydrous sodium sulfate, concentrated on a Vigreux column to 5 mL, and then reduced to a final volume of 300 μL under a gentle stream of nitrogen.

The GC-O system was described in a previous study.^15^ The AEDA was performed in the same manner and using the same parameters as described previously.^15^ Sample distillates were diluted stepwise in diethyl ether from 1:2 up to 1:1024, then subjected to GC-O for evaluation. The original extract was evaluated by three trained panelists, and one trained panelist subsequently carried out the AEDA dilutions.

### Compound Identification by Heart-Cut Two-Dimensional Gas Chromatography with High-Resolution Mass Spectrometry (GC-GC-HRMS)

The identification of 3-methylbut-2-ene-1-thiol (3MBT), 4-methyl-4-sulfanylpentan-2-one (4MSP), 3-sulfanylhexyl acetate (3SHA), 3-sulfanylhexan-1-ol (3SH) and isoborneol was performed by heart-cut two-dimensional gas chromatography with high-resolution mass spectrometry (GC-GC-HRMS). The system consisted of a Trace 1310 gas chromatograph (Thermo Fisher Scientific) equipped with a TriPlus RSH autosampler, a programmed temperature vaporizing (PTV) injector, an FID (250 °C base temperature), and a custom-made sniffing port with a base temperature of 230 °C.^21^ The separation was achieved using a DB-FFAP capillary column (30 m × 0.25 mm i.d., 0.25 μm film thickness; Agilent) with helium as the carrier gas at a constant flow rate of 1.0 mL/min. The injection volume was 1 μL. The initial oven temperature was set at 40 °C and hold for 2 min, followed by a temperature ramp of 6 °C/min to 230 °C, which was held for 5 min. The end of the column was connected to a Deans switch (Trajan; Ringwood, Australia) used for heartcutting. Depending on the programmed timing, analytes were directed via deactivated fused silica capillaries (0.1 mm i.d.) either simultaneously to the FID and the sniffing port or to a second GC column (DB-1701 column, 30 m × 0.25 mm i.d., 0.25 μm film thickness; Agilent) in a second Trace 1310 GC system. Transfer to the secondary system occurred through a heated hose (250 °C) and a liquid nitrogen-cooled trap for analyte reconcentration. The second GC oven operated under the same initial conditions (40 °C, 2 min hold), followed by a temperature ramp of 6 °C/min to 240 °C, with a final hold of 5 min. The outlet of the second column was interfaced with a Q Exactive GC orbitrap mass spectrometer (Thermo Fisher Scientific), operated in the high-resolution EI mode over a scan range of *m*/*z* 35–250. Data acquisition and analysis were performed using Xcalibur software (Thermo Fisher Scientific). Detailed information on the identified compounds can be found in the Supporting Information (Table S3).

### Compound Identification by Gas Chromatography with Mass Spectrometry (GC-MS) and Two-Dimensional Gas Chromatography with Mass Spectrometry (GC-GC-MS)

The identification of Furaneol was done with two-dimensional gas chromatography with mass spectrometry (GC-GC-MS), and that of other compounds was done with a gas chromatograph with mass spectrometry (GC-MS). The instrumental setting for both were described in a previous study,^15^ except for the mass spectrometer of GC-MS was operated with a scan range of *m*/*z* 35–250, while GC-GC-MS was operated in selected ion monitoring mode with individual quantifier ions of each target compound.

### Quantitation of Terpenes and Terpenoids in Dried Hemp Flowers by Gas Chromatography with Flame Ionization Detection (GC-FID)

Given the expected abundance of terpenes and terpenoids in the hemp samples based on AEDA results (Table 1), the quantity of major terpenes and terpenoids was analyzed by a Gas Chromatography (GC) system (Thermo Trace GC Ultra, Brechbühler, Schlieren, Switzerland) with Flame Ionization Detection (FID). Ground dried hemp flower (1 g) was weighed into a plastic centrifuge tube (15 mL), followed by an addition of 6 mL diethyl ether, and 3 mL ultrapure water. Methyl nonanoate (1202 μg) was added as an internal standard corresponding to the expected terpene content. Extraction was performed for 2 h using an overhead shaker, followed by centrifugation at 4000 rpm (3220 g) for 15 min (Eppendorf, Hamburg, Germany). The resulting supernatant was utilized for subsequent quantitation with the GC-FID system, comprising a GC Trace Ultra (Thermo Fisher Scientific, Brechbühler, Schlieren, Switzerland) and a DB-FFAP capillary column (length 30 m, diameter 0.32 mm, film 1 μm) (Agilent Technologies Inc., Basel, Switzerland). The injection volume was 1 μL with a split flow at 50 mL/min and a split ratio of 1:18. The temperature program started at 40 °C and the temperature was held for 3 min, then increased by 8 °C/min to 240 °C and finally held constant for 10 min. Helium was used as carrier gas at a constant flow of 2.8 mL/min. For calibration, three different concentrations of target terpenes and terpenoids were each prepared in diethyl ether and added with the same amount of methyl nonanoate (1202 μg) as in the samples. All calibration solutions were then subjected to GC-FID analysis as mentioned in the instrumental setting. The Supporting Information provides details on the linear regressions used for quantitation. (Table S2)

**Table 1:** Odor-Active Compounds Identified in Aroma Distillates Isolated from Different Hemp Cultivars during AEDA

			retention index on		FD factor *^d^*						
no. *^a^*	odorant^b^	odor quality^c^	FFAP	DB-5	PG071	PG072	PG073	PG074	PG075.1	PG076	ref.
1	ethyl propanoate^e^	fruity, flue-like	943	712	64	16	64	32	128	32	^23^
2	α-pinene	pine-like	1003	930	1024	1024	1024	1024	1024	1024	^12^
3	methyl 3-methylbutanoate *^e^*	fruity	1012	764	128			256	1024		^23^
4	ethyl 2-methylbutanoate	fruity	1023	847	1024	256	64	128	1024	1024	^23^
5	ethyl 3-methylbutanoate	fruity	1053	851	1024	1024	1024		1024	1024	^23^
6	3-methylbut-2-ene-1-thiol	hemp, beer	1085	816	64	32	16	256	256	1024	^9^
7	β-pinene	terpene-like	1092	978	64	32	64	8	256	1024	^12^
8	myrcene	hop-like	1151	991	1024	1024	1024	512	1024	256	^11^
9	1,8-cineole (eucalyptol)	eucalyptus-like	1165	1038	64	256	16	4	16	512	^12^
10	limonene	citrus-like	1190	1033	256	1024	16	128	16	512	^61^
11	β-phellandrene	terpene-like	1234	1035	16			4			^12^
12	unknown	mushroom	1241	1063		32		4			
13	3-methylbutyl 2-methylbutanoate *^e^*	fruity	1267	1100	16						^22^
14	octanal	fruity, citrus-like	1281	941		32					^20^
15	2-acetyl-1-pyrroline *^e^*	popcorn-like,	1322	920	64	32	64	32			^12^
		roasty									
16	4-methyl-4-sulfanylpentan-2-one	sulfury, exotic	1370	934	16	1024	256	64	256	32	^12^
17	3-methoxy-2,5-dimethylpyrazine^e^	earthy	1414	1054	512	256	16	128			^12^
18	acetic acid^e^	vinegar-like	1442	625	64	32	16	128	8	4	^20^
19	3-(methylsulfanyl)propanal (methional)	cooked potato	1453	899	64	256	16	16	64	64	^12^
20	3-isobutyl-2-methoxypyrazine	earthy, bell	1511	1179				32	16	128	^12^
		pepper-like									
21	linalool	citrus-like, floral	1539	1103	256	256	1024	1024	256	256	^12^
22	octan-1-ol *^e^*	citrus-like, green	1553	1167	256		64	128	32	32	^23^
23	β-caryophyllene	moldy	1578	1421	256	16	16	128	4	4	^61^
24	2-acetylpyrazine *^f^*	popcorn	1618	1020				16	128		^23^
25	butanoic acid	sweaty	1620	816	256			128	16		^12^
26	α-humulene	hop-like	1623	1457	64	16	16	128	128	1024	^61^
27	3-methylbutanoic acid	sweaty	1662	857	16	16	64	256	512	64	^23^
28	2-methylbutanoic acid	sweaty	1662	857	16	16	64	256	512	64	^23^
29	isoborneol	moldy	1674	1167	64					64	^22^
30	α-terpineol	floral, citrus-like	1686	1194	64	256	256	128	128		^61^
31	3-sulfanylhexyl acetate	passion fruit	1713	1249	256	32		1024	1024	512	^10^
32	(2*E*)-undec-2-enal^e^,^g^	soapy, metallic	1746	1384	16	8		16	16	128	
33	β-citronellol	perfume, rose-like	1762	1233		32		32	4	4	^62^
34	nerol	rose-like, flowery	1785	1230					128		^63^
35	unknown	passion fruit	1814	1056					16		
36	3-sulfanylhexan-1-ol	tropical fruit,	1829	1125	64	16	16	1024	1024	64	^10^
		exotic									
37	hexanoic acid	sweaty	1839	1012				16			^20^
38	2-methoxyphenol *^g^*	smoky	1854	1095		32					
39	(2*E,*4*E,*6*Z*)-nona-2,4,6-trienal *^f^*	oatmeal-like, sweet	1863	1277	64	256			32		^12^
40	(*E*)-β-ionone	floral, violet-like	1931	1495		32		32	8		^41^
41	*trans*-4,5-epoxy-(2*E*)-dec-2-enal^e^	metallic	2000	1378	64	32	16	8			^12^
42	4-hydroxy-2,5-dimethylfuran-3(2*H*)-one (furaneol)^g^	caramel-like	2031	1067	2			128	1024	128	
43	4-methylphenol (*p*-cresol)	phenolic, smoky	2080	1080	256	1024	32			64	^23^
44	eugenol (4-allyl-2-methoxyphenol)	clove-like, sweet	2162	1358	256	32	32	1024	64	64	^11,39^
45	3-hydroxy-4,5-dimethylfuran-2(5*H*)-one (sotolon)^g^	fenugreek-like,	2179	1108	2	16	16	512	64	16	
		lovage-like									
46	2-methoxy-4-vinylphenol^g^	smoky, clove-like	2194	1316		256	256	256	32	512	
47	methyl anthranilate	sweet	2237	1344	64	64	8	256	256	512	^10^
48	unknown	pepper-like, musty	2256	1720	16	256		128			
49	unknown	peach-like	2398	1653					16		
50	indole	mothball-like	2442	1296	64	64	64	64			^10^
51	3-methyl-1*H*-indole (skatole)	mothball-like	2497	1390	64		64	32			^10^
52	phenylacetic acid^g^	honey-like,	2571	1266		16	8	128	8	16	
		beeswax-like									

a Odorants were numbered according to their retention indices on capillary column FFAP.

b Odorant identified by comparison of its odor quality and intensity at the sniffing port and retention indices on capillaries DB-FFAP, DB-5 as well as mass spectra with data of reference compounds.

c Odor quality perceived at the sniffing port.

d Flavor dilution factor determined by AEDA on capillary FFAP.

e No unequivocal mass spectrum was obtained. Identification is based on the remaining criteria in footnote b.

f Compounds were tentatively identified based on comparison with literature data (odor and retention indices).

g Compounds were reported for the first time in *Cannabis* flowers.

### Statistical Analysis

The F-test for differences between cultivars based on terpene quantitation data was carried out at a significance level of α = 0.05. Statistical analysis and data visualization were done with RStudio (version 4.3.3, Posit PBC).

## Results and Discussion

This study represents the first application of GC-O in combination with AEDA on dried hemp flowers from different cultivars. The result revealed a total of 52 odor-active compounds (Table 1) from various chemical classes as presented in Figure 1. Although many of the compounds identified in this study have been reported before in other hemp and nonhemp materials,^9,12,22,23^ their occurrence as odor-active constituents in dried hemp flowers was reported here for the first time. The distribution of FD factors of each compound across the different samples is visualized in Principal Component Analysis (Figure 3). Beyond terpenes, terpenoids and thiols, compounds such as 2- and 3-methylbutanoic acid, methyl anthranilate, eugenol and 3-hydroxy-4,5-dimethylfuran-2­(5*H*)-one (sotolon) were detected in all cultivars, albeit with varying FD factors. Their occurrence suggests their integral role to the overall odorant profiles of hemp flowers.

**Figure 1:**
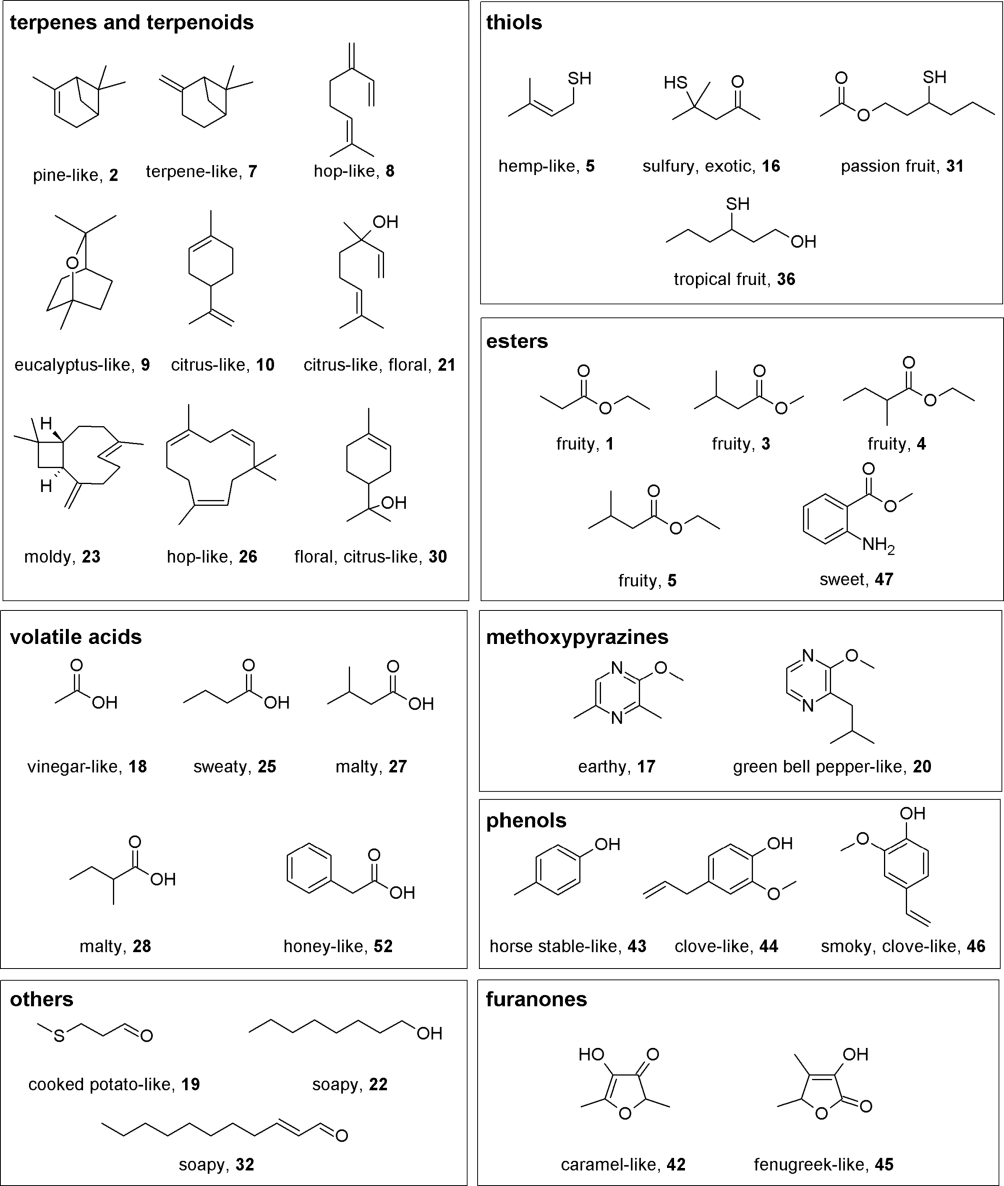
Odor-active compounds of dried hemp flowers, with an FD factor ≥ 128 in at least one of the six analyzed cultivars.

**Figure 2:**
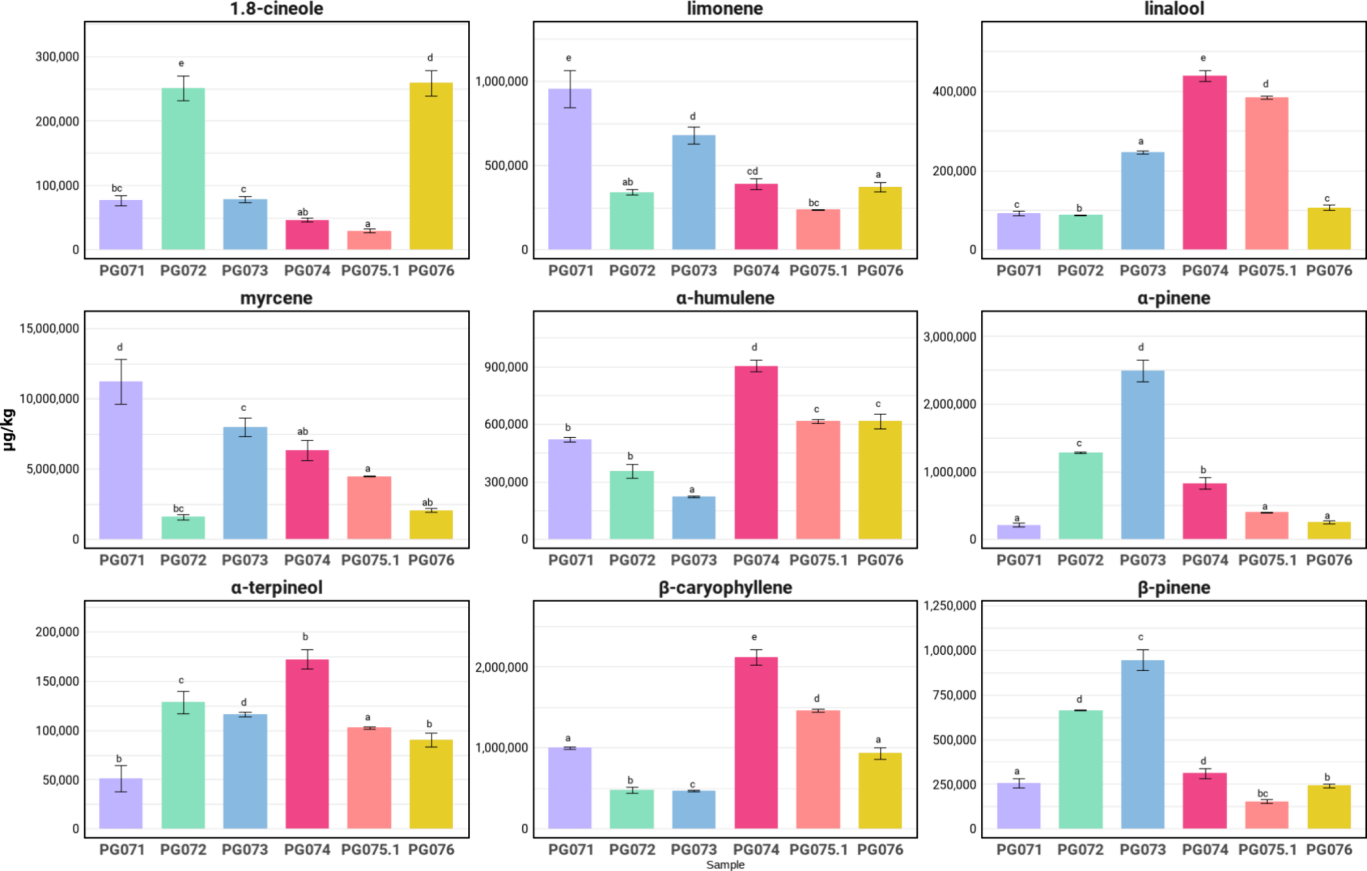
Concentration of odor-active terpenes and terpenoids detected during GC-O analysis in the analyzed dried hemp flowers (different letters indicate a significant difference between the samples for the compound).

**Figure 3:**
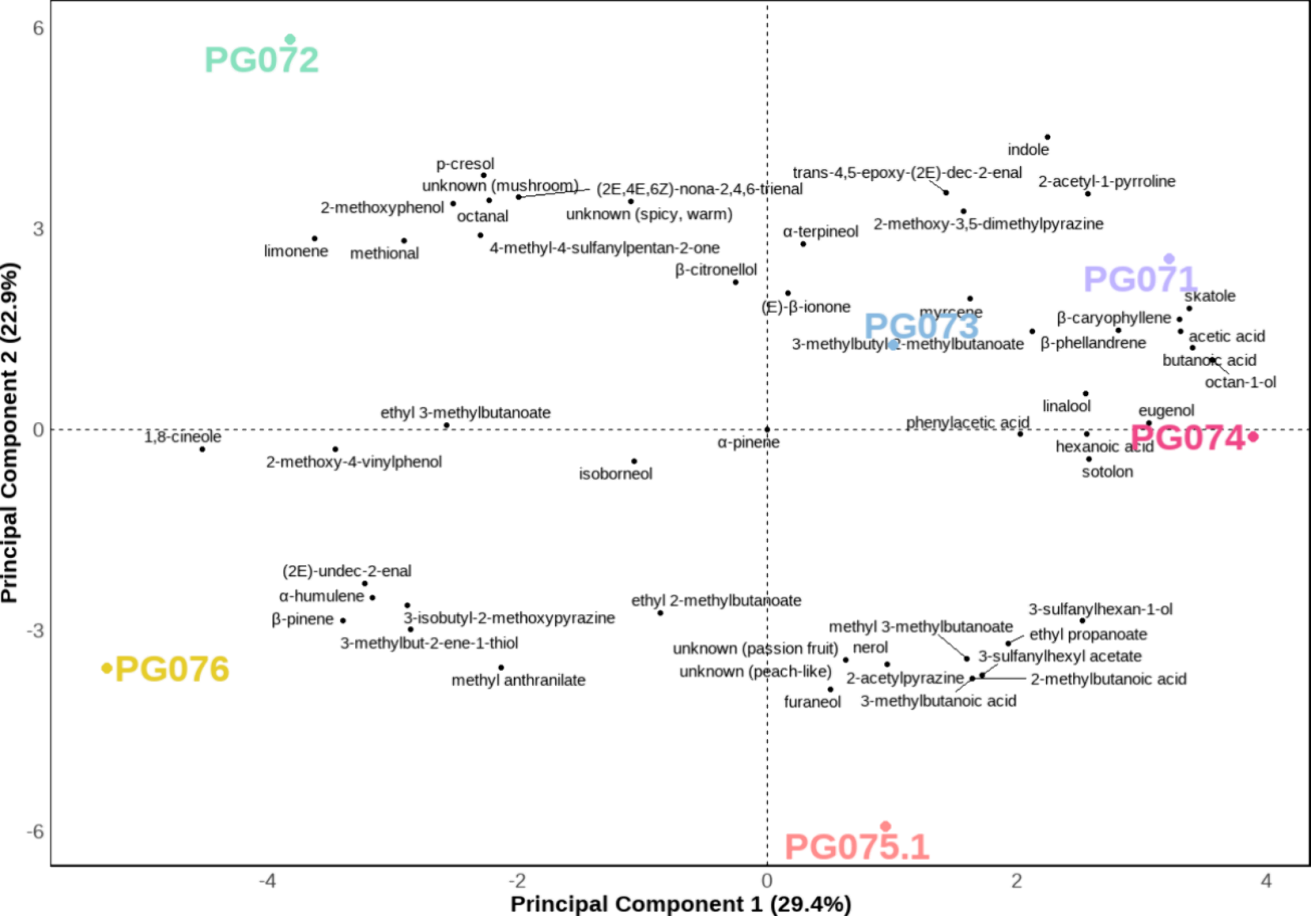
Principal component analysis of odor-active compounds in dried hemp flowers. Odor attributes corresponding to each compound are provided in Table 1.

### Sulfur-Containing Compounds in Dried Hemp Flowers

Sulfur-containing compounds including 3-methylbut-2-ene-1-thiol (3MBT), 4-methyl-4-sulfanylpentan-2-one (4MSP), 3-sulfanylhexyl acetate (3SHA), 3-sulfanylhexan-1-ol (3SH) and 3-(methylsulfanyl)­propanal (methional) were detected in all hemp cultivars at different FD factors. Importantly, the presence of 4MSP as odor-active compounds was unequivocally confirmed for the first time by mass spectral data with GC-GC-HRMS in the present study (Table S3). Methional, known with cooked-potato aroma and to be formed by Strecker degradation from the amino acid methionine,^24^ was detected the most in PG072 (FD 256), while other cultivars displayed only moderate intensities (FD 16–64). Its presence was first reported in fresh hemp flowers.^12^

Regarding the other sulfur-containing compounds, 3MBT showed high FD factors of 256 and 1024 in the PG074, PG075.1 and PG076 cultivars. Meanwhile, 4MSP, 3SHA, and 3SH were detected with high FD factors of 256 to 1024 in the PG074, PG075.1 and PG071 cultivars. These sulfur-containing compounds are known for their strong sensory impact on various plant matrices due to their low odor threshold values.^25^ The compound 3MBT is widely recognized for its contribution to the characteristic “lightstruck” off-flavor in beer,^26^ and “skunky” aroma in durian.^27^ Meanwhile, 4MSP, 3SH, and 3SHA are well-known for their significant roles in tropical fruit aromas such as passion fruit (*Passiflora edulis* f. *flavicarpa*),^28^ guava (*Psidium guajava* L.),^29^ and mango (*Mangifera indica* L.).^30^ These sulfur-containing compounds were also found as odor-active in hops with varied intensities in different cultivars.^16,31^ In Sauvignon Blanc wine, these odorants were known to contribute to the distinct tropical fruit nuances,^32^ therefore they could play a similar role in hemp cultivars that exhibit fruity sensory profiles based on the high FD factors. The presence of 4MSP, 3SH and 3SHA in dried hemp flowers may be linked to precursor pathways involving cysteine and glutathione conjugates, a mechanism that has been well-documented in Sauvignon Blanc wine.^33^ This was also confirmed with hops, where the first identification of 4MSP, 3SH and 3SHA has been linked to nonvolatile cysteine and glutathione-bound precursors.^34^ The detection of thiols in dried hemp aligns with previous findings in cannabis rosin extracts,^9,10^ in which at least their qualitative presence in cannabis has been confirmed. However, their odor activity and relevance to the overall hemp odor had not been analyzed so far. While their exact biosynthetic origin in hemp remains to be elucidated, the consistent detection of 4MSP, 3SH, 3SHA, and 3MBT with high FD factors (256–1024) across multiple cultivars strongly supports their potential role as contributors to the overall aroma profile of dried hemp flowers.

### Esters in Dried Hemp Flowers

The esters identified in dried hemp flowers play a crucial role in shaping the fruity odor sensation, aligning with their well-documented importance in fruits,^35^ hops,^36^ and cannabis.^10^ In this study, AEDAs revealed six odor-active esters, each exhibiting varying FD factors. Among these, ethyl 2-methylbutanoate, ethyl 3-methylbutanoate, and methyl 3-methylbutanoate were found at high FD factors (256–1024), particularly in PG074, PG075.1, PG076, and PG071 cultivars. This aligns with the fruity and citrus descriptors of these cultivars, implying the role of these esters in defining the characteristic fruity notes of hemp materials.^10^

Ethyl propanoate was detected across all cultivars with FD factors ranging from 16 to 256, suggesting its variable but notable contribution. In contrast, 3-methylbutyl 2-methylbutanoate was only present in the PG071 cultivar at an FD factor of 16, indicating a potentially lower olfactory impact compared to other esters. On the other hand, a broader spectrum of esters, including ethyl hexanoate and propyl hexanoate, were detected as abundant volatiles in THC-rich cannabis extracts^10^ from cultivars with exotic odor qualities. However, the impact of these esters on the overall odorant profile was not investigated in the before mentioned study.

### Methyl Anthranilate

Methyl anthranilate, a nitrogen-containing ester known for its grape-like and floral aroma, was detected at high FD factors (256–512) in PG074 and PG076 cultivars, while its presence in other cultivars was significantly lower (FD factor 8–64). These findings suggest that the contribution of this odor-active constituent to the overall odor perception is cultivar-dependent. This compound has previously been quantitated in THC-rich cannabis extracts,^10^ but its impact in dried hemp flowers remains primarily supported by sensory-guided AEDA analysis rather than absolute quantitation. Originally reported in orange flowers, tuberose, and bergamot leaves,^37^ methyl anthranilate has been identified as a key contributor to fruity and floral notes in jasmine tea aroma,^38^ and strawberries.^39^ In jasmine tea it was reported with an odor activity value (OAV) exceeding 1000, highlighting its strong sensory impact. Its relevance in grapes and grape-derived beverages was also documented over a century ago, and it could be demonstrated that the presence of this compound in grapes is highly cultivar-dependent.^32^ This fact mirrors its cultivar-dependent presence in hemp, further reinforcing the role of genetic variation in its formation and expression. Furthermore, due to its low odor threshold (3 μg/kg in water),^40^ methyl anthranilate can significantly contribute to aroma perception even at moderate concentrations. These findings emphasize the need for sensory-guided approaches in hemp aroma research, as nonterpenoid compounds like methyl anthranilate likely play a more substantial role in defining the olfactory character of specific hemp varieties. Moreover, while other anthranilate derivatives have been reported in rosin extracts,^10^ they were not detected in this study and are also uncommon in other plant- and fruit-derived matrices.^25^

### Phenolic Compounds

In this study, 4-methylphenol (*p*-cresol), eugenol, 2-methoxy-4-vinylphenol (4-vinylguaicol), and 2-methoxyphenol were identified with varying FD factors, suggesting a cultivar-dependent influence on hemp aroma. The detection of eugenol and 4-vinylguaiacol at high FD factors (128–1024) across all cultivars highlights their role in defining the sensory perception of dried hemp flowers. Eugenol, which is widely known for its smoky and clove-like aroma, has been detected in Japanese hemp plant,^45^ and cannabis oil,^41,42^ and is part of the aromatic profile of fresh nonhemp cannabis.^20^ As eugenol was not detected in fresh hemp flowers,^12^ its presence in dried samples indicates that postharvest transformations, such as oxidation or enzymatic activity, may enhance its formation. On the other hand, while 4-vinylguaiacol has been previously reported in hops via AEDA,^43^ it has not been detected in hemp materials. This compound is widely recognized in various food matrices, such as beer, for imparting spicy phenolic odor qualities,^44^ and in red wine (*Vitis vinifera* L. ‘Aragonez’) with spicy attributes.^45^ The detection of 4-vinylguaiacol in this study provides the first evidence of its sensory relevance in hemp matrix, suggesting that drying or curing processes may play a role in its generation.

The detection of 4-methylphenol and 2-methoxyphenol further adds complexity to the odor of dried hemp flowers. Both are recognized for their smoky and phenolic aroma and are widely found in smoked foods^46^ and fermented beverages.^47^ While 4-methylphenol was detected in cannabis smoke before,^20^ 2-methoxyphenol was reported for the first time in hemp flowers. Its exclusive presence in PG072 with an FD factor of 32, alongside 4-methylphenol at high intensity based on its high FD factor of 1024, suggests the cultivar-specific aroma contribution of these phenols in hemp.

### Indole and Skatole

Indole and skatole (3-methyl-1*H*-indole) are known for their animalic, and mothball-like odors.^25^ However, they are also used widely in perfumery as flavor enhancers and are abundant in some flowers such as jasmine and orange blossoms.^48^ Indole has been reported as a product of tryptophan degradation during drying and microbial transformation processes.^49^ The detection of indole in this study reinforces the idea that certain postharvest processes, including drying and enzymatic conversions, may enhance its formation in hemp. The study of ice rosin extract suggested that while indole is more common across hemp cultivars, skatole contributes to hemp cultivars with characteristically strong savory descriptions and is typically found in cultivars with intense earthy or musky odor qualities.^10^ However, in contrast to this assumption, skatole was detected in PG071 and PG074 cultivars with FD factors of 32 and 64. These samples were primarily described as fruity, based on their higher FD factors in fruity esters. The co-occurrence of skatole with ethyl 2-methylbutanoate, methyl 3-methylbutanoate, and ethyl propanoate in these cultivars suggests an interplay between fruity and animalic odorants, rather than a direct dominance of skatole over the overall profile. Similarly, indole was detected at FD factors of 32–128, with the highest intensities in PG073 and PG074, suggesting its role in adding floral depth to these cultivars rather than enhancing the fecal and animalic notes. Therefore, their actual impact on the overall hemp odor profiles needs to be further assessed by recombination studies to determine their precise sensory contribution.

### Methoxypyrazines

The presence of methoxypyrazines in dried hemp flowers showed a clear cultivar-dependent occurrence. The compound 3-methoxy-2,5-dimethylpyrazine, known for its earthy aroma,^25^ was found across multiple cultivars, namely PG071 (FD 512), PG072 (FD 256), PG074 (FD 128) and PG073 (FD 16), indicating its broad presence in hemp but at varying intensities. This aligns with the study of fresh hemp flowers, in which 3-methoxy-2,5-dimethylpyrazine was first identified.^12^ Meanwhile, 2-isobutyl-3-methoxypyrazine (earthy, bell-pepper like) was previously detected in only one fresh hemp cultivar^12^ and is now solely identified in PG074 and PG076 cultivars at moderate FD factors (32–128). Interestingly, methoxypyrazines were not detected in rosin extracts,^10^ which most likely can be linked to the low levels of these compounds in different plant matrices and the analytical approach for its detection. This finding reinforces the cultivar-dependent occurrence of this compound in hemp, which will need further investigation by quantitation and aroma reconstitution experiments. In addition, methoxypyrazines are well-documented for their low odor thresholds, making them potent contributors to aroma perception even at trace levels.^40^ The identification of methoxypyrazines in dried hemp flowers underscores the need for the use of sensory-guided techniques, such as the GC-O, to understand their odor profiles on a molecular level. Moreover, the impact of the methoxypyrazines on the odor properties of the selected hemp cultivars require consolidation with reconstitution experiments based on quantitative results.

### Discovery of Odor-Active Furanones in Hemp

The identification of furaneol (4-hydroxy-2,5-dimethylfuran-3­(2*H*)-one) and sotolon (3-hydroxy-4,5-dimethylfuran-2­(5*H*)-one) in dried hemp flowers significantly broadens the known hemp odorant profile, introducing caramel-like and seasoning nuances that have been only partially reported in fresh hemp^12^ or rosin extracts.^10^ This study provides the first confirmation of Furaneol and sotolon as odor-active compounds in dried hemp flowers, indicating a relatively high impact on the overall odor properties of these compounds in some cultivars based on their FD factors. Furaneol was detected exclusively in PG074, PG075.1 (Figure 4) and PG076 cultivars, with FD factors ranging from 128 to 1024, demonstrating a cultivar-dependent expression. The highest FD factor was observed in indoor-cultivated PG075.1 (FD 1024), followed by outdoor-cultivated PG074 (FD 128) and PG076 (FD 128). Furaneol is widely recognized as a key aroma compound in fruits such as strawberries^50,51^ and mangoes,^30^ contributing to ripe, cooked fruit, and caramel-like nuances. The formation of Furaneol occurs via both enzymatic and nonenzymatic pathways, primarily through sugar degradation via Maillard reactions and quinone oxidoreductase (FaQR)-mediated enzymatic conversion as in strawberries.^52^ Hence, its exclusive presence in PG074 and PG075.1 and PG076 hemp cultivars further supports the hypothesis that a similar enzymatic mechanism may be active in certain hemp cultivars, subsequently enhancing the fruity-like odor impression of these cultivars. Furaneol was previously identified in hops^31^ with caramel-like aroma characteristics. While both hops and hemp belong to the Cannabaceae family, their furaneol content may differ based on genetic and environmental factors, with drying and enzymatic activity likely contributing to its higher odor impact in selected hemp cultivars. Furthermore, the absence of furaneol in fresh hemp and its presence in dried flowers supports the assumption that drying may enhance its formation.

**Figure 4:**
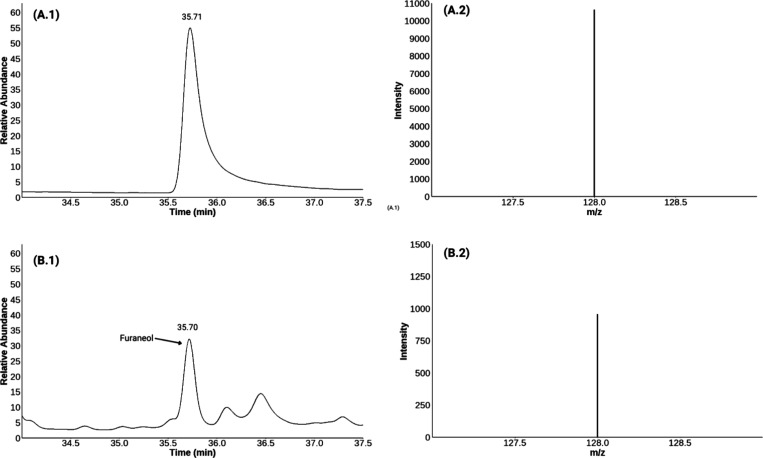
Detection of furaneol in PG074 cultivar via GC-GC-MS analysis: (A.1) Retention time and (A.2) mass spectrum (*m*/*z* 128) of furaneol reference standard; (B.1) Peak in the chromatogram and (B.2) mass spectrum matching furaneol (*m*/*z* 128) in the hemp cultivar extract.

Similarly, sotolon, which was first detected in fresh hemp flowers,^12^ has now been confirmed in dried hemp samples. Sotolon was found in all dried cannabis cultivars, exhibiting the highest FD factor (FD 512) in PG074. The presence of sotolon in dried hemp might also be linked to its formation via the Maillard reaction. Its previous identification in hops^31^ supports its broader relevance in plant-derived aromatic matrices.

### Terpenes and Terpenoid Analysis of Dried Hemp Flowers

In addition to the previously mentioned odorant classes, terpenes and terpenoids—compounds historically emphasized in cannabis and hemp research—were also evaluated in this study. All terpenes and terpenoids detected in the present study were identified in other hemp materials,^12,22,23^ as well as hops.^43^ According to AEDA results, important odor-active terpenes and terpenoids detected with high FD factors across all cultivars included monoterpenes α-pinene (FD 1024), and myrcene (FD 256–1024), as well as the monoterpenoid linalool (FD 256–1024). These compounds had also been reported in prior studies^41,53^ as major terpenes in hemp, primarily based on their high concentrations. Our quantitation results (Figure 2) confirmed their abundance, with myrcene exceeding 10,000,000 μg/kg, particularly in PG071, PG073, and PG074. Similarly, α-pinene was another dominant terpene across all samples, particularly abundant in PG073 and PG072 cultivars with FD factors of 1024. This finding shows a parallel to hemp seed oils, in which α-pinene was detected as key odor-active terpene.^54^ Regarding linalool, though present in lower concentrations compared to myrcene and α-pinene, it exhibited high FD factors (256–1024) across different cultivars. Such a result highlights a strong odor impact due to the compound’s low odor threshold (3.4 μg/kg in oil^36^ and 0.087–2.7 μg/kg in water^25^) —an observation consistent with findings in hops, where linalool was shown to have higher odor activity values despite lower concentrations.^36^ These results confirm that the previously reported abundance of these terpenoids^41,53^ indeed correlates with their sensory relevance, now supported by high FD factors.

Regarding sesquiterpenes, β-caryophyllene and α-humulene were quantitated at notably lower concentrations in dried flowers than in rosin extracts.^10^ However, they still exhibited high FD factors in specific cultivars—namely PG071 (FD 256) and PG074 (FD 128) for β-caryophyllene; and PG076 (FD 1024) for α-humulene. This finding demonstrates its cultivar-specific characteristic as well as its strong odor contribution despite lower abundance. The difference of terpene content between materials also suggests how different processing techniques could influence the concentration of odor-active terpenes. This was observed in dried hemp leaves, which showed decreased β-caryophyllene content with increased drying temperature.^55^

Other terpenoids such as limonene and 1,8-cineole, which were typically associated with citrusy and eucalyptus leaf-like odor qualities, varied significantly between cultivars. Limonene was found in higher amounts in PG071, PG072, and PG076 cultivars (FD 256–1024), whereas in the other cultivars, it had FD factors as low as 4–32. Meanwhile, 1,8-cineole was found in PG072, PG075.1, and PG076 cultivars with high FD factors (FD 256–1024), a finding that contrasts with previous assumptions that it degrades significantly during drying.^55^

Interestingly, some terpenes previously highlighted as abundant in hemp—such as terpinolene and ocimene^56^—were not detected as odor-active in this AEDA analysis. While they may still be present in the matrix, their absence from the sensory-relevant profile underscores the limitations of relying solely on quantitative data. Although quantitation confirmed the high abundance of several terpenes, the FD factor distribution demonstrated that their sensory impact varied widely between cultivars. This highlights that terpene concentration alone cannot fully explain cultivar aroma. These findings reinforce the importance of applying sensory-guided techniques like GC-O and AEDA, alongside quantitative analysis to better understand the contribution of individual compounds to the overall aroma.

### Other Compounds

The current findings also expand the known aroma complexity of dried hemp beyond the previously studied terpenoids and sulfur compounds, confirming the presence of additional odorants that enrich the odor complexity of hemp.

The role of volatile acids in hemp aroma warrants attention. While previous studies linked octanoic and decanoic acids in rosin extracts to cheesy and astringent attributes,^11^ this study identified 2-methylbutanoic acid, 3-methylbutanoic acid, butanoic acid, and acetic acid with consistently high FD factors (FD 128–512) in PG074, PG075.1 and PG076 cultivars. These volatile acids can be found in fruits such as apricots,^57^ strawberries,^50^ and wine,^33^ where they act as important aroma contributors. In wine, they are known to interact with esters and other volatiles to intensify fruity notes.^58^ Therefore, the presence of volatile acids at higher FD factors in hemp cultivars with fruity sensory attributes suggests that these acids may also enhance the fruity perception of these cultivars. Notably, acetic acid was previously identified only in cannabis smoke^20^ and was detected in dried hemp flowers for the first time. The absence of acetic acid in fresh flowers^12^ and rosin extracts^10^ indicates its possible formation via postharvest oxidation or microbial activity.

Finally, the cultivar-dependent occurrence of additional compounds such as (*E*)-β-ionone, (2*E*)-undec-2-enal and phenylacetic acid (PAA) further highlights the diversity of the composition of hemp aroma. The two latter compounds were detected for the first time in hemp flowers. (2*E*)-Undec-2-enal exhibited the highest intensity in the PG076 cultivar (FD 128), whereas its presence in other cultivars was limited to much lower FD factors (FD 8–16). The (2*E*)-undec-2-enal formation pathway could be linked to lipid oxidation, as previously shown in olive oil.^59^ On the other hand, phenylacetic acid was observed at an FD factor of 128 only in PG074, while other cultivars were observed with lower intensity (FD 8–32). In plants, phenylacetic acid is primarily synthesized from the amino acid phenylalanine.^60^ The detection of phenylacetic acid in dried hemp flowers could derive from a similar mechanism with the oxidation of phenylacetaldehyde that was already present in fresh cannabis.^12^ Lastly, (*E*)-β-ionone appeared to be more cultivar-dependent than the other two odorants, when it was only found in PG072 and two PG074 and PG075.1 cultivars with modest FD factors of 8 and 32. A similar finding was also observed in hop,^16^ in which (*E*)-β-ionone was perceived at FD factors of 8 and 16. The occurrence of these additional odorants further differentiates the sensory profiles of specific cultivars from other hemp varieties, highlighting the complexity of hemp’s aroma compounds.

### Molecular Composition of the Scent of the Analyzed Hemp Flowers

As a complementary visualization of odor-active compounds distribution across cultivars, Principal Component Analysis (Figure 3) was performed based on FD factors obtained through AEDA, highlighting differences in sensory profiles and the molecular compositions between cultivars. The first two principal components (PC1 and PC2) explained 29.4% and 22.9% of the total variance, respectively. PG074 and PG075.1 are grouped together in the lower right quadrant, being associated with fruity and tropical-smelling odorants such as esters, acids, Furaneol, and thiols, reflecting their strawberry-like aroma. PG071 and PG073 were also nearby fruity esters, but PG071 associated more closely with other sweaty-smelling odorants like 3-methylbutanoic acid and butanoic acid. Meanwhile, PG073 aligns more with β-caryophyllene, skatole, and linalool, reflecting earthy, herbal, and slightly sweet characteristics. PG076 clustered separately, driven by citrusy and terpene-like compounds such as β-pinene and α-humulene. PG072 was positioned in the upper left region and associated with compounds like *p*-cresol, and 2-methoxyphenol, suggesting its turpentine-like and spicy description.

In summary, the present study provides the first comprehensive sensory-guided investigation into the composition of the odor-active compounds of dried hemp flowers, revealing the intricate interplay between terpenes, esters, sulfur compounds, and previously underexplored odorants such as phenolic compounds, volatile acids, and furanones. Through AEDA analysis, 52 odor-active compounds have been identified. There are 38 odorants that had not been reported in dried hemp flowers before and six that were identified in hemp material for the first time. The presence of these new odor-active components further supports the idea that certain odorants may be formed or released during drying and curing. Future research is needed to explore how enzymatic or oxidative pathways contribute to these transformations. The findings of the study underline the importance of the use of sensory-guided techniques such as the GC-O in combination with AEDA as an unequivocal step to understanding odor impressions on a molecular level. To fully understand the contribution of these odor-active odorants, future studies need to be performed with the aim to quantitate these key odorants by stable isotope dilution assays (SIDA) to accurately determine their dose-over-threshold (DoT) values. Furthermore, reconstitution and omission studies will be necessary to assess the precise impact of individual compounds and their synergies in the hemp aroma space. Ultimately, these insights lay the groundwork for breeding strategies aimed at enhancing specific aroma attributes in hemp cultivars. By deepening the knowledge of cannabis secondary metabolism, targeted breeding efforts could optimize the production of desirable odorant compounds, catering to distinct market preferences in food, fragrance, and cannabis-based consumer products.

## Supplementary Material



## Notes

(Table S1) Dried hemp sample information, (Table S2) parameters used in the quantitation of terpenes and terpenoids, and (Table S3) mass spectral data of compounds identified via GC-GC-HRMS (PDF)

## Abbreviations Used

3MBT: 3-methylbut-2-ene-1-thiol
3SH: 3-sulfanylhexan-1-ol
3SHA: 3-sulfanylhexyl acetate
4MSP: 4-methyl-4-sulfanylpentan-2-one
AEDA: aroma extract dilution analysis
CBD: cannabidiol
DoT: dose over threshold
EI: electron ionization
FD: flavor dilution
GC-GC-HRMS: heart-cut two-dimensional gas chromatography with high-resolution mass spectrometry
GC-GC-MS: two-dimensional gas chromatography–mass spectrometry
GC-MS: gas chromatography–mass spectrometry
GC-O: gas chromatography-olfactometry
OAV: odor activity value
OT: odor threshold
PAA: phenylacetic acid
PCA: principal component analysis
RI: retention index
SAFE: solvent-assisted flavor evaporation
THC: tetrahydrocannabinol
