# Supplementary material for: The Plant of Many Scents: Unraveling the Odorant Composition of Selected CBD Hemp Cultivars
Source: J Agric Food Chem. 2025 Sep 10;73(38):24314–25. doi: 10.1021/acs.jafc.5c07208 (PMC12464981; doi:10.1021/acs.jafc.5c07208)
Supplement: Supplementary file 1 [file jf5c07208_si_001.pdf]

Supporting Information

The plant of many scents: unraveling the odorant composition of selected CBD hemp cultivars

Thi Khanh Linh Tran,[1] Tatiana Avellaneda,[1] Amandine André,[1] Elodie Gillich,[1] Martin Steinhaus,[3] Dániel Árpád Carrera,[2] Leron Katsir,[2] Irene Chetschik.[1]*

[1]Zurich University of Applied Sciences (ZHAW), Life Sciences and Facility Management, 8820 Wädenswil, Switzerland.

[2]Puregene AG, 4314 Zeiningen, Switzerland.

[3]Leibniz Institute for Food Systems Biology at the Technical University of Munich (Leibniz-LSB@TUM), 85354 Freising, Germany.

---

*Corresponding author:

Irene Chetschik

Phone: +41 58 934 5651

Fax: +41 58 934 5001

E-mail: irene.chetschik@zhaw.ch

Table S1. Dried Hemp Samples Information

no.	sample code	cultivar description	growth environment	post-harvest	CBD (% w/w)[a]	THC (% w/w)[b]
1	PG701	Bubble gum	glass house	freeze-dried	16.6	0.7
2	PG702	Amnesia	glass house	freeze-dried	11.2	0.3
3	PG703	Harlequin	glass house	freeze-dried	14.2	0.4
4	PG704	Strawberry 1	glass house	freeze-dried	13.4	0.5
5	PG705.1	Strawberry 2	field	freeze-dried	14.4	0.6
6	PG706	Lemon	glass house	freeze-dried	< 11	< 1

[a,b]Measured and provided by Puregene AG.

Table S2. Parameters Used in the Quantitation of Terpenes

target compound	internal standard	calibration line	R^2
α-pinene	methyl nonanoate	y = 0.9241x - 0.0048	1.0000
β-pinene	methyl nonanoate	y = 1.0217x + 0.0016	0.9999
myrcene	methyl nonanoate	y = 0.7235x + 0.1313	0.9994
limonene	methyl nonanoate	y = 1.0286x - 0.0077	0.9981
1,8-cineole	methyl nonanoate	y = 0.7527x - 0.0004	0.9998
β-caryophyllene	methyl nonanoate	y = 1.2070x - 0.0534	1.0000
linalool	methyl nonanoate	y = 1.1014x + 0.0049	0.9981
α-humulene	methyl nonanoate	y = 0.7229x + 0.0149	0.9996
α-terpineol	methyl nonanoate	y = 1.0385x - 0.0005	0.9993

Table S3. Mass Spectral Data of Identified Compounds as Obtained by GC–GC–HRMS

compound	RI-FFAP	RI-DB-5	MS-EI data[c]
3-methylbut-2-ene-1-thiol[a]	1099	878	41.03846(50); 67.05431(40); 68.06209 (100); 69.06986(80); 102.04984(10)
4-methyl-4-sulfanlylpentan-2-one[a]	1375	934	43.0178 (100); 75.02364 (8); 132.06075 (10)
3-sulfanylhexyl acetate[b]	1713	1249	43.01788(25); 55.05438(60); 67.05438(50); 83.03427(45); 88.03427(100); 101.04215(40); 118.06142(35)
3-sulfanyl-hexan-1-ol[a]	1840	1125	54.0000-56.0000; 66.00000-83.00000; 81.0000-83.0000;99.00000-101.00000
isoborneol[a]	1672	1170	93.06989 (22); 95.08552 (100); 121.10141 (13); 136.12494 (5)

[a]olfactory detection on RI-FFAP and RI-DB-5 & comparison with reference standard, verification of the compound with MS-EI spectral data
[b]olfactory detection on RI-FFAP and RI-DB-5 & comparison with reference standard, verification of the compound was done by screening the respective main MS-EI spectrum fragments
[c]*m*/*z* (% relative intensity cf. base peak)
